# Repurposing Chloroquine Against Multiple Diseases With Special Attention to SARS-CoV-2 and Associated Toxicity

**DOI:** 10.3389/fphar.2021.576093

**Published:** 2021-04-12

**Authors:** Siya Kamat, Madhuree Kumari

**Affiliations:** Department of Biochemistry, Indian Institute of Science, Bengaluru, India

**Keywords:** SARS-CoV-2, pH-dependent, autophagy, immunomodulatory, antiviral mechanism, toxicity

## Abstract

Chloroquine and its derivatives have been used since ages to treat malaria and have also been approved by the FDA to treat autoimmune diseases. The drug employs pH-dependent inhibition of functioning and signalling of the endosome, lysosome and trans-Golgi network, immunomodulatory actions, inhibition of autophagy and interference with receptor binding to treat cancer and many viral diseases. The ongoing pandemic of COVID-19 has brought the whole world on the knees, seeking an urgent hunt for an anti-SARS-CoV-2 drug. Chloroquine has shown to inhibit receptor binding of the viral particles, interferes with their replication and inhibits “cytokine storm”. Though multiple modes of actions have been employed by chloroquine against multiple diseases, viral diseases can provide an added advantage to establish the anti–SARS-CoV-2 mechanism, the *in vitro* and *in vivo* trials against SARS-CoV-2 have yielded mixed results. The toxicological effects and dosage optimization of chloroquine have been studied for many diseases, though it needs a proper evaluation again as chloroquine is also associated with several toxicities. Moreover, the drug is inexpensive and is readily available in many countries. Though much of the hope has been created by chloroquine and its derivatives against multiple diseases, repurposing it against SARS-CoV-2 requires large scale, collaborative, randomized and unbiased clinical trials to avoid false promises. This review summarizes the use and the mechanism of chloroquine against multiple diseases, its side-effects, mechanisms and the different clinical trials ongoing against “COVID-19”.

## Introduction

Chloroquine, commonly known for the anti-malarial applications has evolved gradually as a magic medicine, effective against many diseases including rheumatoid arthritis (RA), systemic lupus erythematosus (SLE), multiple types of cancer and viruses. It has also been a molecule of choice among research community for studying the mechanism of autophagy, nanoparticles internalization, endocytosis and interlinked role of multiple signalling pathways in various diseases including cancer and autophagy (Pelt et al., 2018; Varisli et al., 2019).

Recent onset of the Coronavirus Disease-2019, a pandemic which has put the world on its knees, has again brought this “age-old drug” chloroquine and its derivatives into bright limelight. The disease has already spread worldwide and has killed more than 9,534,437 of the world population (Coronavirus Death Toll and Trends-Worldmeter, n.d.) and is still affecting millions. Multiple drugs are being tested, and the research community leaves no stone unturned to come up with an effective vaccine or drug to treat this wide-spread disease. Chloroquine and its derivatives have also emerged as a potential drug for effective treatment of this novel coronavirus (Smith et al., 2020; Touret and Lamballerie, 2020). Other potential drugs being tested for COVID-19 are remdesivir (GS-5734), lopinavir;ritonavir, Interferon alfacon-1 in conjunction with corticosteroids and Ribavirin in conjunction with corticosteroids (Smith et al., 2020; Wang M. et al., 2020). However, none of the drugs being researched has been approved by the World Health Organization (WHO) for the treatment of COVID-19 till now, keeping the room open for further research on chloroquine and the derivatives.

4-N-(7-chloroquinolin-4-yl)-1-N, 1-N-diethylpentane-1, 4-diamine, commonly known as chloroquine is a 4-aminoquinoline approved by FDA for treatment of malaria and inflammation–related diseases. It is a colorless and odourless crystal with a molecular mass of 319.9g/mol and available as a generic medicine (PubChem ID: 2719). Chloroquine is an inexpensive, water-soluble, weakly basic tertiary amine, which at physiological pH (7.2–7.4) is highly membrane permeable. However, inside the acidic organelles, it gets protonated and accumulates, raising the pH of the respective organelle. It can interfere with all the pH-dependent signalling and functioning of the endosome, lysosome, Golgi network, phagosome, and autophagosomes (Weyerhäuser et al., 2018). However, due to some side-effects of chloroquine, several derivatives, including hydroxychloroquine have been synthesized with similar efficacy but reduced toxicity.

**FIGURE 1:**
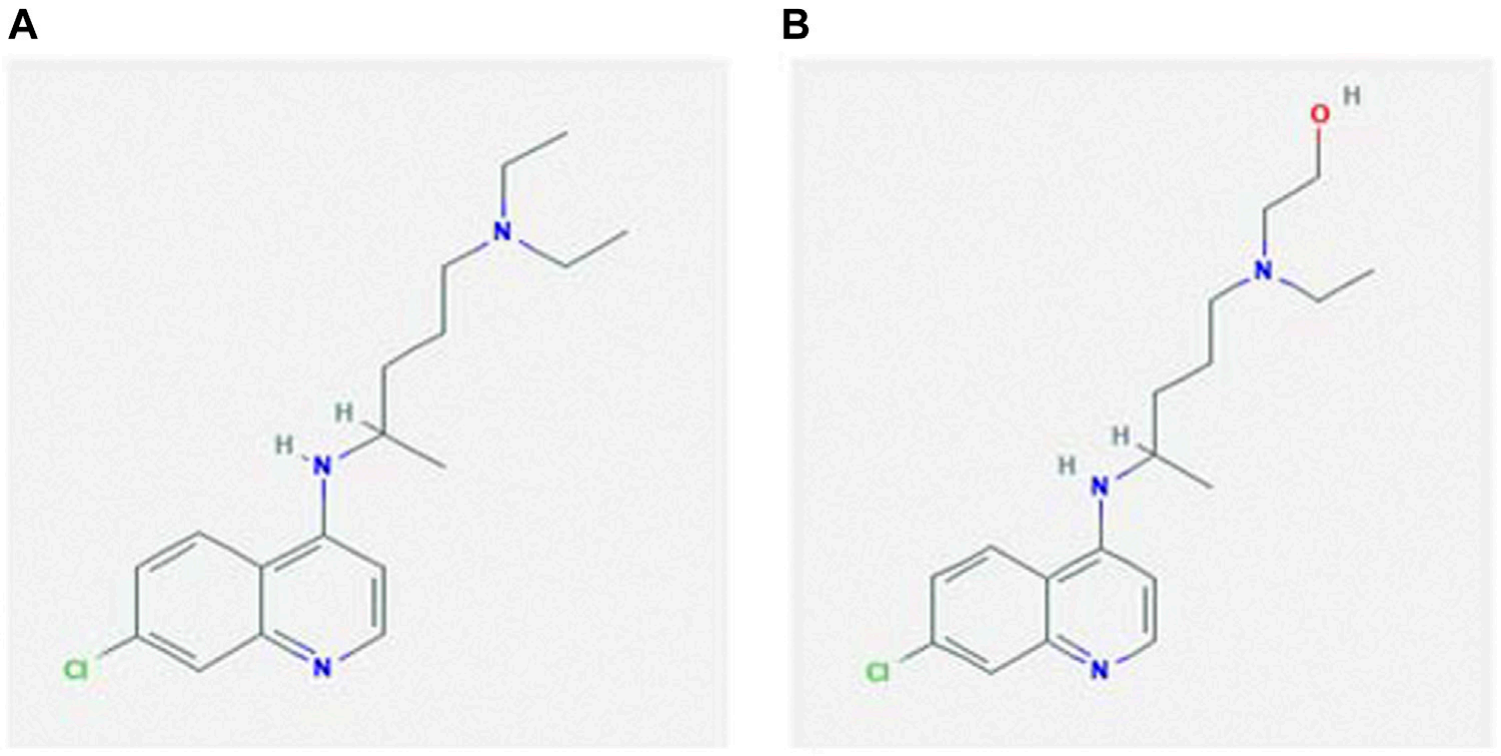
Chemical structure of **(A)** chloroquine and **(B)** hydroxychloroquine (National Center for Biotechnology Information, 2004).

Chloroquine and its derivatives (Figure 1), emerging as one of the most probable drugs alone or in combination against the battle of COVID-19, needs a detailed compilation and review so that the mechanisms elucidated by them against multiple diseases can be understood and co-related or used for the further vaccine and drug development for COVID-19. This review summarises chloroquine’s journey, from being an anti-malarial drug to a magic bullet against multiple diseases, its good and evil, results of clinical trials obtained so far and the future aspects, it holds along with its drawbacks as prophylaxis or drug to fight COVID-19.

## Chloroquine as an Anti-malaria Drug

### Mechanism of Haemoglobin Degradation Inside the Human Body by the Malaria Parasite

To understand, how chloroquine inhibits malarial parasite, it is important to know the mechanism employed by *Plasmodium* sp. to hijack erythrocytes and use haemoglobin for their energy requirements. The *Plasmodium* sp. has a specialized acidic organelle known as digestive vacuole (DV) for degrading haemoglobin for its energy requirements following a cascade of protease activities (Pandey and Chauhan, 1998). The by-product of haemoglobin digestion is heme. Heme, when bound in haemoglobin is in the non-toxic ferrous form (Fe^2+^), but when free, it converts into very toxic ferric form (Fe^3+^) (Francis et al., 1994). To avoid toxicity, the parasite must evolve machinery to get rid of toxic heme, which is achieved by crystallization of heme called “hemozoin” or “malarial pigment” (Coronado et al., 2014). The formation of hemozoin takes place at considerably low pH where two heme units are linked together by iron carboxylate bonds. This unusual linkage is important for the synthesis and growth of an ordered insoluble crystal (Bohle et al., 1997; Coronado et al., 2014). Histidine rich protein (HRP) plays a vital role in the biocrystallization of hemozoin (Coronado et al., 2014). The hemozoin formed does not only detoxify the heme pigment for parasite but also adversely affects the human immune system, especially macrophages (Schwarzer et al., 2003).

### How Chloroquine Works Against the Malaria Parasite

There were several theories proposed regarding the mode of action of chloroquine to kill malaria pathogen as DNA binding agent (Parker and Irvin, 1952) protein synthesis inhibitor (Surolia and Padmanaban, 1991) polyamine metabolism inhibitor (Slater, 1993) and inhibitor of hemozoin crystallization (Orjih, 1997; Gorka et al., 2013). Most of the studies have shown chloroquine as a potent inhibitor of hemozoin crystallization. Sullivan. et al. (1996) postulated that chloroquine inhibits hemozoin formation by inhibiting HRP II. Again, Sullivan. et al. (1998) in their study concluded that chloroquine blocked the polymerization of free heme released during haemoglobin proteolysis in intraerythrocytic *P. falciparum*. Later in a review, Sullivan (2017) summarized that quinolones block every step of toxic heme crystal growth. DVs are acidic organelles with pH 5.0, where chloroquine can diffuse inside easily. However, the acidic pH yields diprotonation of the drug, inhibiting its movement out of the DV. The trapped diprotonic chloroquine inhibits the crystal growth of hemozoin, toxifying the malaria pathogen (Goldberg, 1993). Pandey and Tekwani et al. (1997) in their study established that chloroquine initiates a reverse reaction of conversion of hemozoin to monomeric heme (ferriprotoporphyrin IX) after interaction with malarial hemozoin, also termed as termed “hemozoin depolymerization”.

### Developing Resistance by *Plasmodium* sp. Against Chloroquine and Alternative Strategies

Developing resistance by *Plasmodium* sp*.* against chloroquine attributes to a point mutation in the genes coding for the chloroquine resistance transporter (PfCRT) present in DV (Martin et al., 2009; Chinappi et al., 2010). This protein avoids the accumulation of chloroquine by facilitating the efflux of the diprotonic chloroquine. However, the action of protein as a channel or a carrier is still debatable. Chinappi et al. (2010) in their study proposed that the protein acts as a carrier to exclude out both mono and diprotonic chloroquine. Reiling et al. (2018) proposed that pharmacological responses of sensitive and resistant malaria parasite towards chloroquine are also different.

Different strategies including alternative drugs, derivatives of chloroquine and combinational drug therapies have been used to combat the chloroquine-resistant malarial parasite. Clindamycin in combination with quinine was successfully used for the treatment of uncomplicated multidrug-resistant *P. falciparum* malaria in Thai patients (Pukrittayakamee et al., 2000). Artesunate-atovaquone-proguanil combination has proven successful for the treatment of the similar case of malaria (van Vugt et al., 2002). Primaquine, mefloquine, artesunate and artemisinins are some of the drugs used in the treatment of resistant malaria in India (Kalra et al., 2002). Treatment of chloroquine-resistant malaria using a combination of pyrimethamine, berberine, tetracycline or cotrimoxazole has been used successfully to treat chloroquine-resistant malaria in Africa (Sheng et al., 1997).

## Chloroquine as an Anti-Rheumatoid Arthritis and Lupus Erythematosus drug

RA and LE are autoimmune diseases, where healthy tissues are attacked by the hyper-immune system causing inflammatory responses. RA is mainly characterized by pain, inflammation and stiffness around the joints, whereas LE is characterized in the early phase with arthritis, skin lesions, inflammation around the lungs and kidneys. Rhupus, is a syndrome which presents symptoms associated with both RA and LE (Macfarlane and Manzel, 1998; Thome et al., 2013).

Both chloroquine (CQ) and 4-hydroxychloroquine (HCQ) are extensively used as immune-modulators to treat RA and LE. There are evidence of both, pH-dependent and pH-independent role of chloroquine and its derivatives to inhibit the generation of autoantibodies and reducing the secretion of inflammatory cytokines (Macfarlane and Manzel, 1998; Thome et al., 2013).

CQ and HCQ both can enter acidic endosome and lysosome, remain there as CQ^+^ and CQ^++^, elevate their pH from 4.5 to 6.0, and interfere with their functions (Mindell, 2012). By interfering with endosome functions, it inhibits TLR7 and nine signalling and thus inhibits dendritic cell maturation. By changing the acidity of lysosome of antigen-presenting cells, CQ and HCQ, inhibits the presentation of the major histocompatibility (MHC) complex peptides to T cells, thus inhibiting the production of T helper cells and cytokines (Thome et al., 2013; Ponticelli and Moroni, 2017). It also inhibits calcium-dependent signalling, toll-like receptor signalling pathways, and iron metabolism in macrophages, thus suppressing production of IL-6, IL-1 and tumor necrosis factor-alpha (TNF-α) (Ponticelli and Moroni, 2017; Schrezenmeier and Dörner, 2020). Rand et al. (2008) in their experiment using atomic force microscopy (AFM) observed that hydroxychloroquine interferes with binding of antiphospholipid antibody–β2-glycoprotein I complexes to phospholipid bilayers, thus lowering down inflammation. Oh et al. (2016) concluded in their study that chloroquine reduces inflammation through p21-mediated suppression of T cell proliferation and Th1 cell differentiation.

Though the development of resistance against disease-modifying anti-rheumatic drugs (DMARDs) including chloroquine, has not been studied much, the role of ATP binding cassette (ABC) proteins responsible for drug efflux cannot be neglected (Jansen et al., 2003). A better understanding is needed in this field to establish alternative strategies and drug combination therapy for RA and LE.

## Chloroquine as an Anti-Cancer Drug

### How Chloroquine Works Against Cancer

Inhibition of cancer cell growth by chloroquine is a complex process. Table 1 summarizes the multi-ranged effects of chloroquine on multiple types of cancer cells. The primary mechanism employed by chloroquine and its derivative is inhibition of autophagy during cancer cell death. The pH-dependent accumulation of chloroquine inside lysosome leads to impairment of autophagosome degradation and thus inhibition of autophagy (Mauthe et al., 2018). It is also known to generate endoplasmic stress, lysosome and mitochondrial membrane depolarization in a reactive oxygen species (ROS) dependent manner, thus increasing apoptosis (Ganguli et al., 2014; Alam et al., 2016). Though chloroquine alone is not sufficient to depolarise membrane potential; it is generally used to sensitize chemo or radiotherapy, in an autophagy-dependent or independent manner (Maycotte et al., 2012; Makowska et al., 2016; Zhu et al., 2019). However, there are some severe kidney and organ injuries have also been reported after the use of chloroquine as the sensitizer to chemo and radiotherapy (Kimura et al., 2013).

**TABLE 1:** Examples of chloroquine used in treatment of cancer.

S. No	Name of drug	Type of Cancer Cell	Concentration of chloroquine	Mechanism	Reference
1	Chloroquine with C2 ceramide	Lung Cancer H460 and H1299 Cells	10 µM	Inhibition of autophagosome maturation and degradation during autophagy progression	Chou et al. (2019)
2	Chloroquine with Luteolin	Squamous Cell Carcinoma Cells	50 µM	Blocked autophagy	Verschooten et al. (2012)
3	Chloroquine as an adjuvant	Glioma cells	5–20 µM	Blocked autophagy and modulated several metabolic pathways, deficient DNA repair	Weyerhäuser et al. (2018)
4	Chloroquine	Bladder cancer cells	20 µM	Inhibition of cholesterol metabolism	King et al. (2016)
5	Chloroquine and GX15-070	Pancreatic cancer cells	20 µM	Blocked autophagy	Wang et al. (2014)
6	Chloroquine	Rat sarcoma	1–100 µM	Sensetized cells by inhibition of DNA repair and loss of mitochondrial potential	Eng et al. (2016)
7	Chloroquine with temozolomide	Glioma cells	5–20 µM	Sensetizing glioma cells by autophagy inhibition	Yan et al. (2016)
8	Hydroxycholoroquine with phytosterol	Lung cancer cell	20–120 µM	Autophagy inhibition	Elshazly et al. (2020)
9	Chloroquine with Tenovin-6	Gastric cancer	25–50 µM	Autophagy inhibition	Ke et al. (2020)
10	Hydroxychloroquine	HeLa cells	60 μg/ml	Loss of lysosome and mitochondrial membrane potential	Boya et al. (2003)
11	Chloroquine and NVP-BEZ235	Neuroblastoma cells	0–120 µM	Lysosome -mitochondria cross talk	Seitz et al. (2013)
12	Chloroquine	Pancreatic cancer	0.5–100 μg/ml	Inhibition of neutrophil extracellular traps	Boone et al. (2018)
13	Chloroquine	Prostate cancer	10–20 µM	Induces Par-4 response	Rangnekar (2019)
14	Chloroquine	Bladder cancer	10 µM	Enhances the radiosensitivity by inhibiting autophagy	Wang et al. (2018)
15	Chloroquine and oxaliplatin	Pancreatic cancer	—	modulating activity of cytosolic HMGB1	Lee et al. (2018)

Recent studies have revealed that chloroquine is also able to interfere with different metabolic pathways, including cholesterol, glucose, amino acids, and mitochondria metabolism (Weyerhäuser et al., 2018).

**FIGURE 2:**
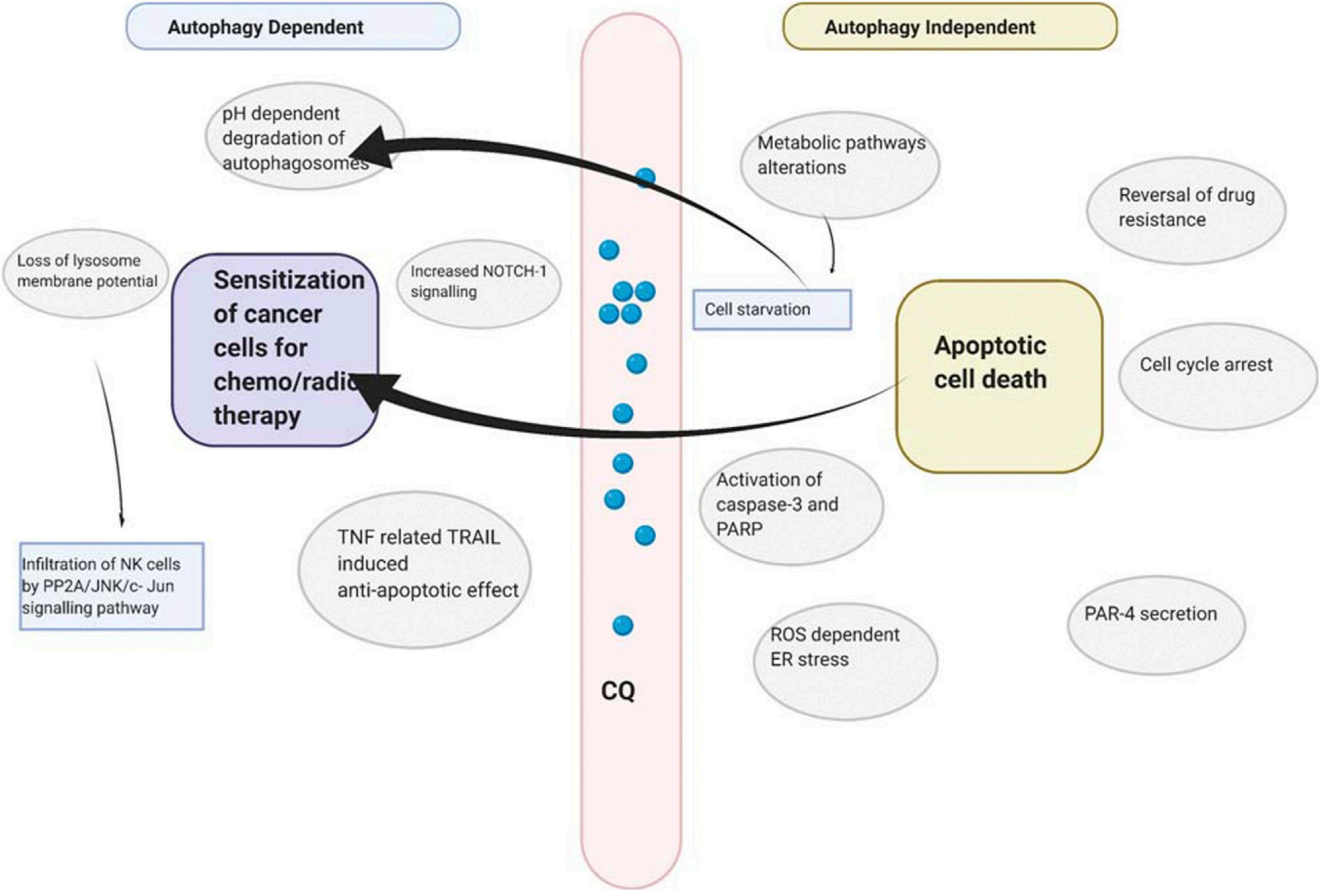
Probable mechanism employed by chloroquine to kill cancer cells.

Chloroquine is also used to treat multidrug-resistant cancer by blocking drug extrusion by interfering with the ATP-binding cassette (ABC) transporter family and other transmembrane protein related to drug resistance (Szakács et al., 2006). A summary of mechanisms employed by chloroquine has been illustrated in Figure 2.

**FIGURE 3:**
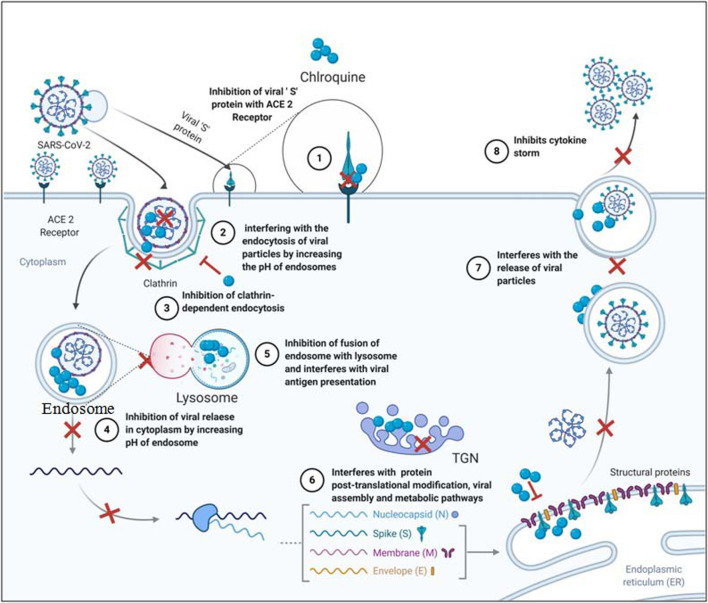
Probable mechanism employed by chloroquine to inhibit SARS-CoV-2.

## Chloroquine Against Bacterial and Fungal Diseases

Generally, in response to intracellular bacterial or fungal pathogens, the first-line antimicrobial defence is initiated by the phagocytes. After being internalised by the phagocyte, a phagosome forms which further fuses with lysosomes. Through oxygen dependent and independent mechanisms, the bacteria are killed. This acidifies the phagolysosome to pH 4.5 and activates lysosomal enzymes. Several intracellular pathogenic bacteria and fungi evade this line of defence through different mechanisms such as, they lack the lysosomal pathway (ex. *Bartonella* sp.), escape before the fusion of phagosome and lysosome and survive in the cytosolic region (ex. *Shigella* sp, *Rickettsia* sp.), block lysosomal fusion and multiply in the phagosome (ex. *Chlamydia* sp, *Salmonella* sp*, Mycobacterium* sp*, Yersinia* sp), resistance to survival in phagolysosome (*Coxiella burnetii*, *Tropheryma whipplei*). Chloroquine treatment inhibits the growth of these intracellular pathogens by pH dependent iron deprivation and neutralising the phagolysosomal pH (Rolain et al., 2017). Lagier et al. (2014) reported the bactericidal combination treatment of doxycycline and hydroxychloroquine against the classic *Tropheryma whipplei* caused Whipple’s disease. The authors confirmed the effectivity of the combination treatment through *in vitro* studies and clinical trials (Lagier et al., 2014). Q fever, caused by *Coxiella burnetii* infection manifests into a severe complication of endocarditis. A combination of doxycycline and chloroquine derivates has been reported to reduce the mortality rate and is a prominent therapeutic intervention for Q fever. The mechanism of action is under investigation; however, it can be presumed that chloroquine increases the lysosomal pH and enhances the antibacterial activity of doxycycline (Alegre et al., 2012; Lagier et al., 2014).

## Chloroquine Against Viral Diseases with Special Attention to SARS-CoV-2

### Viral Pathogenesis in Human With Special Attention to SARS-CoV-2

The catastrophic impact of viral diseases on human has been observed since ages. From Spanish flu to COVID-19, humankind has always struggled to make a way out of socio-economic burden slapped by viral pathogens. The COVID-19 pandemic crisis has worsened the economic and health condition worldwide to such a level that had not been observed in the last 70years (https://www.un.org/development/desa/dspd/2020/04/social-impact-of-covid-19/). The COVID-19 outbreak is detrimental to old age, immuno-suppressive people, and a significant economic burden on indigenous and poor people.

Each virus has a different virulence factor, and the pathological consequences also differ from virus to virus. The knowledge of viral pathogenesis is neither accurate nor complete for most viral infections, especially for SARS-CoV-2. Novel SARS-CoV-2 is an enveloped single-stranded RNA virus belonging to the family Coronaviridae (Zheng, 2020) responsible for ongoing pandemic COVID-19. The main symptoms of this disease include fever, cough and fatigue, and it can lead to severe complications, having a mortality rate of 5.7% (Lechien et al., 2020) 50% of the COVID-19 positive patients are asymptomatic. The main symptoms in the early stages are headache (70%) loss of smell, and nasal obstruction. Cough, fever and dyspnoea are a sign of late infection (8–10days) (Lechien et al., 2020; Sajna and Kamat, 2020).

Discussing complete progress details about the viral pathogenesis will be beyond this review, however, in general, pathogenic virus and in particular, SARS-CoV-2 follows the following events to cause an infection.

a. Entry inside the cells in an endocytosis-dependent or independent manner.

Most of the human viruses follow an endocytosis-dependent entry inside the cells. The envelope spike glycoprotein of SARS-CoV and SARS-CoV-2 binds to the angiotensin-converting enzyme 2 (ACE2) receptor on target cells to facilitate entry (Li et al., 2020; Zhang et al., 2020). The spike “S” protein is responsible for the ACE2 receptor binding, whereas the cellular serine protease TMPRSS2 is required to prime the “S” protein (Hoffmann et al., 2020).

Xu et al. (2020) in their study found that the 3D structure of the receptor-binding domain in both the viruses is identical. SARS-CoV followed direct membrane fusion between the virus and plasma membrane as well as clathrin-dependent and -independent endocytosis mediated entry inside target cells (Wang et al., 2008; Kuba et al., 2010).

b. Viral replication inside target cells.

The replication mechanism of SARS-CoV and SARS-CoV2 is also found to be similar (Caly et al., 2020). After entry inside the target cells, the virus’s RNA genome is released in the cytoplasm, translated, and posttranslational modifications occur in endoplasmic-reticulum or Golgi apparatus. After the assembly of RNA and nucleocapsid proteins, the replicated virus particles are released by membrane fusion (Li et al., 2020).

c. Escaping immune surveillance

Most of the viral diseases survive inside human by escaping immune surveillance. Viruses of the family Coronaviridae are no exception. During the initial infection, the SARS-CoV-2 delays type 1 IFN production and avoids the recognition by pattern recognition receptors (PRRs), allowing uncontrolled viral replication, activating pro-inflammatory cytokines triggering “cytokine storm” (Huang et al., 2005; Li et al., 2020; Rothan and Byrareddy, 2020). Further, activation of specific Th1/Th17 enhances the inflammatory responses.

SARS-CoV-2 escapes activation of adaptive immunity by interfering differentiation and function of dendritic cells and defensins and a severe decrease in CD4^+^ and CD8^+^ T cells (Li X. et al., 2020; Li G. et al., 2020).

### How Chloroquine Works Against Viral Diseases

Chloroquine acts as a potent anti-viral agent by implying several mechanisms which have been listed in Table 2. The anti-viral mechanisms of chloroquine can further be exploited to develop it as a therapeutic agent against SARS-CoV-2.

**TABLE 2:** Examples of chloroquine used against viral diseases.

S. no	Drug	Viral Disease	Concentration	Mechanism	Reference
1	Chloroquine	Human Coronavirus OC43	15 mg of chloroquine per kg of body weight	Not established	Keyaerts et al. (2009)
2	Chloroquine	SARS-CoV	10–50 µM	Elevations of endosomal pH, terminal glycosylation of the cellular receptor, angiotensinconverting enzyme 2	Vincent et al. (2005)
3	Hydroxyferroquine Derivatives	SARS-CoV	IC_50_- 0.3–1 μg/ml	Not established	Biot et al. (2006)
4	Chloroquine, 7-8-dihydroneopterin	SARS-CoV, MERS-CoV	EC_50_ 3–8mol/L, 4 mg/kg per day	Endosomal acidification	Al-Bari (2017)
5	Chloroquine	MERS-CoV	EC_50_ of 3 µM	Inhibited replication	Liang et al. (2018)
6	Chloroquine	Zika virus	20 mg/kg of body weight	Protection against ZIKV-induced inflammatory changes	Li et al. (2017)
7	Chloroquine	Ebola virus	10 µM	Lysosome acidification. Was able to inhibit *in vitro* but failed *in vivo*	Dowall et al. (2015)
8	Chloroquine	Zika virus	5–40 µM	obstructs fusion of the flaviviral envelope protein with the endosomal membrane	Shiryaev et al. (2017)
9	Chloroquine	Herpes simplex virus	15 µM	Interacts with endocytic viral entry	Dai et al. (2018)
10	Chloroquine	Influenza A virus	60 µM	Blocking autophagy	Calderon et al. (2019)
11	Chloroquine	Zika virus	0–300 μm/l	Blocking autophagy	Zhang et al. (2019)
14	Hydroxy-chloroquine	Dengue virus	0–100 µM	Activating ROS and a MAVS mediated host IFN anti-viral pathway	Wang et al. (2015)
15	Hydroxy-chloroquine	Influenza A virus	3–30 µM	Blocking autophagy	Yan et al. (2013)
16	Chloroquine	Influenza A virus	500 mg/day for 1week	Disrupts pH-dependent structural changes in viral-synthesized proteins	Paton et al. (2011)
17	Chloroquine	HIV	100 µM	Interferes with innate immunity-induced immune hyperactivation	Martinson et al. (2010)
18	Hydroxy-chloroquine	HIV	20 µM	Apoptosis in the memory T-cell compartment by inhibiting autophagy	van Loosdregt et al. (2013)
19	Hydroxy-chloroquine	HIV	—	Induction of a defect in the maturation of the viral envelope glycoprotein gp120	Tsai et al. (1990)
20	Chloroquine	Chikungunya	250 mg/day	Not established	Chopra et al. (2014)
21	Chloroquine	Prion (scrapie-infected neuroblastoma (ScN2a))	100 µM	Acidification of lysosome	Supattapone et al. (1999)
22	SGI-1027 (Derivative of Chloroquine)	Creutzfeldt-Jakob disease	0–1μm/L	reduce PrP^Sc^ formation via direct coupling with PrPC in prion-infected cells	Kim et al. (2019)
23	Chloroquine	Influenza B virus	0–10 µM	lysosomotropic alkalinizing agents (LAAs) and calcium modulators (CMs)	Marois et al. (2014)
24	Chloroquine	Human Papilloma Virus (HPV)	10 µM	Autophagy inhibition, inhibited the up-regulation of PD-L2	Baruah et al. (2019)
25	Chloroquine	Grass carp reovirus (GCRV)	50–400 µM	Inhibition of Lysosomal acidification	Wang et al. (2016)
26	Chloroquine and hydroxyl-chloroquine	Human Papilloma Virus (HPV) (Cutaneous warts)	400 mg/day	Inhibition of Lysosomal acidification	Bhushan et al. (2014)
27	Hydroxy-chloroquine	SARS-CoV-2	EC_50_ = 1.13μM	Interfering with the glycosylation of cellular receptors and endosome alkylatiation	Wang et al. (2020)
28	Hydroxy-chloroquine	SARS-CoV-2	400 mg given twice daily for 1day, followed by 200mg twice daily for 4 more	Not established	Yao et al. (2020)
29	Hydroxy-chloroquine	SARS-CoV-2	CC_50_ 249.50 μM	Inhibition of endocytosis	Liu et al. (2020)
30	Hydroxy-chloroquine and azithromycin	SARS-CoV-2	600 mg of hydroxyl-chloroquine daily	Not established	Gautret et al. (2020)
31	Chloroquine and hydroxyl-chloroquine	SARS-CoV-2	In silico study	Inhibition of viral S protein to bind with gangliosides	Fantini et al. (2020)
32	Hydroxy-chloroquine	SARS-CoV-2	400 mg given twice daily for 1 day, followed by 200 mg twice daily for 4 more days	Not established	Clementi et al. (2020)
33	Chloroquine and hydroxyl-chloroquine	SARS-CoV-2	IC_50_ 46 and 11μM	Not established	Weston et al. (2020)
34	Hydroxy-chloroquine and azithromycin	SARS-CoV-2	1, 2 and 5 μM for 78 hydroxy-chloroquine and 2, 5 and 10 μM for azithromycin	Not established	Andreani et al. (2020)
35	Chloroquine	SARS-CoV-2	EC_50_ of 1.13 μM	Not established	Gao et al. (2020)

### Probable Mechanisms of Chloroquine Against SARS-CoV-2

Though studies are still ongoing on chloroquine as an inhibitor of SARS-CoV-2, the plausible mechanisms known from its use against various diseases can provide a substantial ground for further research and development of chloroquine as a potential drug against COVID-19. Multiple modes of actions of chloroquine against SARS-CoV-2 are as follows:

1. Inhibition of viral entry inside the target cells

Chloroquine can inhibit the binding of viral spike glycoprotein with ACE2 receptor on target cells to inhibit their entry. Chloroquine has shown potent inhibition of sugar modifying enzymes or glycosyltransferases and quinone reductase which have been involved in sialic acid biosynthesis of ACE2 receptor (Kwiek et al., 2004; Devaux et al., 2020). Wu et al. (2020) in their docking studies showed that chloroquine can potentially target Nsp3b or E-channel with the docking mfScores of–130.355 and–107.889, respectively, though experimental results are yet to be verified.

SARS-CoV-2 particles significantly resemble the nanoparticles with a size of 60–140nm and are spherical. Nanoparticles are known to exhibit their desired results by cell internalization (Kumari et al., 2017) which can effectively be inhibited by chloroquine. Chloroquine inhibits nanoparticles internalization by suppression of phosphatidylinositol binding clathrin assembly protein (PICALM), thus inhibiting clathrin-dependent endocytosis (Hu et al., 2020). The same principle can be applied for stopping the internalization of SARS-CoV-2 particles inside the target cells. Chloroquine can also play a vital role in interfering with the endocytosis of viral particles by increasing the pH of endosomes which has been explained earlier (Touret and Lamballerie, 2020). Interaction of TMPRSS2 with the ACE2 receptor is essential for facilitating SARS-CoV-2 entry (Matsuyama et al., 2020). Application of chloroquine with a known serine protease inhibitor can weaken the viral entry inside the cells (Markus et al., 2020). Serine protease inhibitor camostat mesylate has been observed to blocks TMPRSS2 activity in SARS-CoV-2 (Hoffmann et al., 2020).

2. Inhibition of viral replication and posttranslational modifications (PTM)

Chloroquine inhibits acidification of endosome and lysosome, stalling the virus inside endosomes and inhibiting the release of the viral RNA genome in the cytosol. Inhibition of lysosome acidification further hampers the fusion of endosome with the lysosome and upstream trafficking essential for viral replication (Devaux et al., 2020; Hu et al., 2020; Wang et al., 2020).

Inhibition of acidification further continues to work in favour of chloroquine against SARS-CoV-2 as it inhibits posttranslational modification in trans Golgi network (TGN). Lack of low pH in TGN interferes with functional proteases and glycosyl-transferases resulting in impaired PTM or non-infectious viral particles (Devaux et al., 2020; Touret and Lamballerie, 2020).

3. Inhibition of autophagy

Many of the human viruses employ autophagy for their replication inside the target cells (Table 2) (Yan et al., 2013; Calderon et al., 2019; Zhang et al., 2019). Though the role of autophagy in the proper functioning of SARS-CoV-2 is still under investigation, several results claim that autophagy is crucial for SARS-CoVs replication (Brest et al., 2020; Yang and Shen, 2020). Prentice et al. (2004) demonstrated the critical role of endogenous LC-3, a protein marker for autophagosomes in the replication of SARS-CoV. Chloroquine, being a well-established autophagy inhibitor can be a potential candidate for suppression of COVID-19.

4. Immuno-modulator and inhibition of “cytokine storm”

Chloroquine is widely used for the treatment of RA and SE based upon its immune-modulatory properties. As discussed in earlier sections, chloroquine inhibits pH-dependent toll-like signalling pathway in the endosome and inhibits the inflammatory response “cytokine storm”. The inhibition of toll-like signalling pathway prevents the recognition of viral antigen by dendritic cells (Devaux et al., 2020). It also enhances cytotoxic CD8^+^ T cell responses against viral antigens and exports soluble antibodies into the cytosol of the dendritic cell to fight the viral antigen.

Chloroquine interferes with viral antigen presentation via the lysosomal pathway and thus inhibits MHC II recognition of antigen, modulating the elevation of inflammatory responses (Kearney, 2020). Inhibition of TNFα, TNF α receptors and TNF α signalling by chloroquine plays a vital role in the suppression of “cytokine storm” (Touret and Lamballerie, 2020).

5. Interference with the metabolic pathways

As it is already known from the use of chloroquine against glioblastoma, this can regulate metabolic pathways especially lipid metabolisms in cells. Lipid metabolic pathways play an important role in viral entry and replication inside the target cells. SARS-CoV-2 infection interfered with the regulation of lipid metabolism with the higher concentration of free fatty acids, lysophosphatidylcholine, lysophosphatidylethanolamine, and phosphatidylglyceroland significant lower concentration of total cholesterol (TC), HDL-cholesterol and LDL-cholesterol levels in serum (Hu et al., 2020; Zheng et al., 2020). As observed during treatment of glioblastoma and SLE, chloroquine can also regulate metabolic pathways during SARS-CoV-2 infection as its therapeutic mode of action.

## Chloroquine Against Other Diseases

Apart from being an FDA approved anti-malaria, anti-RA, and anti-LE drug, chloroquine has been investigated against several other prevalent medical conditions. Table 3 summarises the use of chloroquine in the treatment of other diseases and its mode of action.

**TABLE 3:** Examples of chloroquine used in treatment of multiple diseases.

S. no	Name of Disease	Name of the drug	Mode of Action	Reference
1	Graft-versus-host disease (GVHD)	Chloroquine	Alterations in T-cell cytokine production	Schultz et al. (2002)
2	Graft-versus-host disease (GVHD)	Hydroxychloroquine	Immunomodulator	Gilman et al. (2012)
3	Porphyria cutanea tarda (PCT)	Chloroquine	Release of bound hepatic porphyrin and its rapid elimination	Scholnick et al. (1973)
4	Porphyria cutanea tarda (PCT)	Hydroxychloroquine	Interaction with large amounts of porphyrins	Singal et al. (2012)
5	Porphyria cutanea tarda (PCT)	Hydroxychloroquine	Interaction with large amounts of porphyrins	Singal et al. (2019)
6	Sarcoidosis	Chloroquine and hydroxychloroquine	Suppression of the granulomtous inflammation	Beegle et al. (2013)
7	Sarcoidosis	Chloroquine and hydroxychloroquine	Not established	Sharma (1998)
8	Granuloma annulare	Chloroquine and hydroxychloroquine	Not established	Masmoudi et al. (2006)
9	Granuloma annulare	Chloroquine and hydroxychloroquine	Anti-inflammatory responses	Rodriguez-Caruncho and Marsol (2014)
10	Lichen planus	Chloroquine	Not established	De Argila, et al. (1997)
11	Lichen planus	Hydroxychloroquine	Lowering the expression of regulatory T cells	Zhu et al. (2014)
12	Urticaria vasculitis	Chloroquine	Not established	Loricera et al. (2014)
13	Osteoporosis	Chloroquine	Decreases the intracellular pH in mature osteoclasts and stimulates cholesterol uptake	Both et al. (2018)
14	Osteoporosis	Chloroquine	Not established	Stapley (2001)
15	Avascular Necrosis	Chloroquine	Immunomodulator	Roberts et al. (2018)
16	Diabetes Type II	Chloroquine	Alterations in insulin metabolism and signaling through cellular receptors	Hage et al. (2014)
17	Diabetes Type II	Chloroquine	ATM activation	McGill et al. (2019)
18	Diabetes Type II	Chloroquine	Reduction in lysosomal degradation of the internal insulin-insulin receptor	Wondafrash et al. (2020)
19	Cardiovascular Diseases	Chloroquine and hydroxychloroquine	Decreased levels of total cholesterol, triglycerides, and low-density lipoprotein-cholesterol (LDL-c)	Liu et al. (2018)
20	Thrombosis	Chloroquine	Inhibition of neutrophil extracellular traps	Boya et al. (2003)
21	Thrombosis	Chloroquine	Disaggregation of ADP-stimulated platelets and inhibition of thrombin-and A23187-induced aggregation	Jancinová et al. (1994)
22	Glanders and melioidosis	Chloroquine	pH Alkalinization of type 6 Secretion System 1 and Multinucleated Giant Cells	Chua et al. (2016)
23	Q fever	Chloroquine	Restore intracellular pH allowing antibiotic efficacy for *Coxiella burnetii*	Calza et al. (2002)
24	Whipple’s disease	Chloroquine	The downregulation of tumour necrosis factor-a expression	Lagier et al. (2014)
25	Whipple’s disease	Hydroxychloroquine	Not established	Alegre et al. (2012)
26	Giardiasis	Hydroxychloroquine	Not established	Escobedo et al. (2014)
27	Antiphospholipid syndrome	Hydroxychloroquine	Reduces antiphospholipid antibodies levels	Nuri et al. (2017)
28	Antiphospholipid syndrome	Hydroxychloroquine	Reduces antiphospholipid antibodies levels	Wang and Lim (2016)
29	Antifungal activity against *H. capsulatum* and *C. neoforman*	Chloroquine and hydroxychloroquine	Inhibition of phagolysosomal fusion and by expression of a unique endogenous H+-ATPase	Rolain et al. (2017)
30	Antifungal activity against *Aspergillus niger*	Hydroxychloroquine	pH-dependent iron deprivation	Keshavarzi et al. (2016)

## Chloroquine Toxicity

Chloroquine and the derivatives while using as an anti-malaria drugs in Mâncio Lima, Acre, Brazil, caused itching, stinging sensation, epigastric pain, and diarrhoea (Braga et al., 2015). It was explained by enhanced production of IgE, degranulation of mast cells and basophils creating allergy like reaction. However, severe side-effects including mental confusion, seizures, coma, and cardiovascular symptom, was not reported. Adedapo et al. (2009) observed that higher dosage of chlorpheniramine plus chloroquine (10 mg/kg daily for 3 days) in children below 5 years caused drowsiness and lower respiratory rates, though no additional benefits were obtained. Chloroquine is known to induce concentration-dependent cytotoxicity, which should always be optimized before finalizing the dosage.

Though optimized dosage and short-term treatment of RA with CQ and HCQ was considered safe, long-term use of CQ in a 64-year-old woman resulted in both restrictive and hypertrophic myocardiopathy auricular-ventricular blocks due to long term pH alteration in the lysosome (Cervera et al., 2001). Kelly et al. (1990) also focussed on the narrow margin between therapeutic uses of chloroquine against RA and the chloroquine poisoning. They reported the death of a 12-month-old infant after receiving 300 mg of chloroquine. They also highlighted the different dose optimization of chloroquine for adults and infants. Scherbel et al., (1965) in their clinical trials found that out of 741 patients treated with chloroquine derivative for SLE, 31-68% developed retinopathy and marked destruction of rod and con cells. However, no clear relationship between chloroquine dosage and retinal toxicity could be established. Lane et al., (2020) performed a multinational retrospective study to evaluate the risk of HCQ alone and in combination with azithromycin in 956,374 RA patients (18 years and above). It was observed that a 30-days dose of HCQ demonstrated no risk of adverse events. However, long term use of HCQ alone increased cardiovascular mortality. A combination of HCQ and azithromycin elevated the risk of heart failure even in the short term. Therefore, the authors suggest a careful consideration of benefit:risk ratio when starting HCQ treatment (Lane et al., 2020).

It is difficult to estimate the frequency of adverse events because many cases have been reported in more than one publication and lack a criterion for diagnosis (Scherbel et al., 1965). Hence, it is recommended to evaluate cardiac health with ECG and ophthalmological examinations for 6 months before prescribing a long-term treatment with chloroquine (Scherbel et al., 1965; Kelly et al., 1990; Cervera et al., 2001).

Chloroquine, as a chemotherapeutic agent against cancer, can act as a double-edged sword. It not only sensitizes the cancer cells but also the normal cells by blocking autophagy and impairing lysosome and endosomes' function (Kimura et al., 2013; Choi et al., 2018). Kidney is the most critically affected organ during chemotherapy with chloroquine with significant nephrotoxicity (Klionsky et al., 2016; Wang B. et al., 2020). Evangelisti et al. (2015) also reviewed the substantial side effects of chloroquine while treating acute leukaemia. Repurposing chloroquine against cancer was generally considered safe for short term treatment with optimized dosage. However, patients suffering from glucose-6-dehydrogenase deficiency, impaired hepatic and kidney diseases should always be cautious while practising chloroquine and derivatives as a chemotherapeutic agent (Verbaanderd et al., 2017). A clinical trial (ClinicalTrials.gov ID NCT04201457) by “Pediatric Brain Tumor Consortium” is ongoing to assess the safety and benefit of adding hydroxychloroquine to dabrafenib and/or trametinib in children with recurrent or progressive low grade or high-grade brain tumor with specific genetic mutations whose results are waited in February 2025 (https://clinicaltrials.gov/ct2/show/results/NCT04201457).

The standard and optimized dose of chloroquine as prophylaxis and during treatment of diseases do not bear significant toxicity, however, long term use of higher concentration of chloroquine can result in severe toxicity (Table 4). Use of less toxic derivatives such as hydroxychloroquine, optimized dosage, nanoencapsulation of the drug and combinational therapies have been used for reducing chloroquine toxicity and increasing efficacy (Amolegbe et al., 2018; Lima et al., 2018).

**TABLE 4:** Toxic effects of chloroquine.

S. no	Drug	Toxicity^a^	Concentration/Duration/Dosage	Reference
1	Chloroquine	Ocular toxicity	250 mg of chloroquine per day for 6 monthe-14 years	Puavilai et al. (1999)
2	Hydroxy-chloroquine	Retinopathy	Inadequately Weight Adjusted Dosage	Arendt and Gerding (2017)
3	Hydroxy-chloroquine	Retinopathy	≥5 mg per kilogram per day	Zaidi et al. (2019)
4	Chloroquine	NeuromytoToxicity	200–500 mg per day for 7m-16 years	Estes et al. (1987)
5	Chloroquine	Neurotoxicity	Variable concentration in culture media	Bruinink et al. (1991)
6	Chloroquine	Renal toxicity	50 mg^−1^kg for 4weeks	Wang et al. (2020)
7	Chloroquine	Renal toxicity	—	Wiwanitkit (2015)
8	Chloroquine and hyrdoxy-chloroquine	Cutaneous toxicity	200–500 mg/day for 7 years	Martin-Garcia et al. (2003)
9	Hyrdoxy-chloroquine	Stevens-Johnson syndrome	40 mg/day for 2 weeks	Leckie and Rees (2002)
10	Amodiaquine	Hematological toxicity	—	Parhizgar and Tahghighi (2017)
11	Chloroquine	Leukemia	For several months	Nagaratnam et al. (1978)
12	Chloroquine	Hepatotoxicity	Combination of proguanil 200 mg and chloroquine 100 mg	Wielgo-Polanin et al. (2005)
13	Hydroxy-chloroquine	Ototoxicity	Hydroxychloroquine 5 mg/kg/day (400 mg/day)	Fernandes et al. (2018)
14	Chloroquine	Cardiotoxicity	250 mg/day for 9 years	Teixeira et al. (2002)
15	chloroquine	Alveolitis	For two weeks	Mitja et al. (2000)
16	Chloroquine and hydroxyl-chloroquine	Myopathy	3.5 mg/kg/day for chloroquine and 6.5 mg/kg/day for hydroxychloroquine for 40.4 months	Casado et al. (2006)
17	Chloroquine	Pruritus	—	Aghahowa et al. (2010)

^a^ The frequency of chloroquine induced toxicity and adverse effects is difficult to estimate due to lack of common methods of diagnosis and metrics of evaluation.

Hypokalemia toxicity is commonly observed in chloroquine and hydroxychloroquine overdose due to the intracellular shift of potassium. Clemessy et al. (1995), performed a retrospective study of 191 cases of chloroquine toxicity in which the initial clinical features included, gastro-intestinal disturbances, neurological impairment, and respiratory symptoms and eventual blockage of potassium channels contributed to hypokalaemia (Clemessy et al., 1995).

Neuropsychiatric manifestations including depression, psychosis, insomnia, agitation have also been reported due to acute or chronic use of chloroquine and hydroxychloroquine (Juurlink, 2020).

Hematologic toxicities are attributed to its long half-life in plasma which leads to accumulation in the blood cells. Lymphopenia, eosinophilia is typically observed immunologically mediated idiosyncratic drug reactions (Juurlink, 2020).

Prolongation of the QT interval due to both chloroquine and hydroxychloroquine has also been observed since the drugs interfere with vascular repolarization. It was observed that after a dose of 600 mg QTc increases 6.1 and 28 ms after a dose of 1,200 mg. However, this effect varied in younger age groups. In response to this treatment in COVID-19 patients, ventricular tachycardia and ventricular fibrillation and mortality were observed potentially due to the overdosage. Hence, in a COVID-19 setting FDA cautions the use of HCQ or CQ, but not in cases of malaria, lupus and RA (https://www.fda.gov/drugs/drug-safety-and-availability/fda-cautions-against-use-hydroxychloroquine-or-chloroquine-covid-19-outside-hospital-setting-or). In severe COVID-19 cases where azithromycin was co-prescribed in combination with either chloroquine and hydroxychloroquine, Molina et al. (2020) reported no evidence of rapid anti-viral clearance or any associated clinical benefit in only 11 patients, possibly because they had significant comorbidities such as obesity, cancer, HIV infection (Molina et al., 2020). However, in a retrospective study 3,737 COVID-19 patients treated with hydroxychloroquine/azithromycin and other regimens in Lagier et al. (2020) observed otherwise. Along with 3 days early treatment of HCQ-azithromycin resulting in faster viral load reduction, no cases of *torsade de pointe* or sudden death were observed. This could be because the patients belonged to mean age of 45 years, the treatment was initiated very early with a dosage of 200 mg of oral HCQ, three times daily for ten days and 500 mg of oral azithromycin on day 1 followed by 250 mg daily for the next four days, respectively.

Sacher et al., (2020) propose a pragmatic approach to mitigate the cardiac risk in the COVID-19 setting. The authors propose a cardiac algorithm for critically reviewing patient’s clinal history (use of other drugs that may extend QT interval, levels of serum K^+^, creatinine, and a recent 30 s ECG). In cases of QT intervals >500 ms, the authors recommend that a QT-prolonging drug should not be prescribed (Sacher et al., 2020).

Some rare immunologically mediated adverse reactions including Stevens-Johnson syndrome, toxic epidermal necrolysis, DRESS (drug reaction with eosinophilia and systemic symptoms), have been implicated in chloroquine and hydroxychloroquine treatment against viral diseases. Although rare, these conditions turn into serious entities when accompanied by liver or kidney injury (Juurlink, 2020).

## Results of Clinical Trials Done so far With Chloroquine Against COVID-19

Currently, there are multiple clinical trials underway to investigate the potential use of hydroxychloroquine and chloroquine alone or in combination against SARS-CoV-2 (Table 5). Some of the *in vitro* and *in vivo* results obtained with chloroquine and hydroxychloroquine supported their anti-viral role against SARS-CoV-2, (Andreani et al., 2020; Clementi et al., 2020; Fantini et al., 2020; Gao et al., 2020; Gautret et al., 2020; Liu et al., 2020; Weston et al., 2020; Yao et al., 2020), however, results of Gautret et al. (2020) faced severe criticism because of small sample size, overruling type I errors, inconsistency between study protocols and lack of blinding and a control arm even though the treatment resulted in viral load reduction. It is also very important to reproduce the *in vitro* results obtained with chloroquine in the *in vivo* studies and clinical trials to establish it as a safe and effective anti-SARS-CoV-2 drug.

**TABLE 5:** Ongoing clinical trials to evaluate the potential of chloroquine and hydroxychloroquine against SARS-CoV-2.

S. no	Clinical trial no	Location	Details	Dosage	Current status	Results	Reference
1	NCT04328493 (April 7, 2020)	Vietnam	Randomized trial, 250 participants	250 mg chloroquine tablet	Phase 2	Expected by April 1, 2022	https://clinicaltrials.gov/ct2/show/NCT04328493
				Adult ≥53 kg: 4 tabs			
				Adult 45–52 kg: 3.5 tabs			
				Adult <38 kg: 2.5 tabs			
2	NCT04358068 (May 1, 2020)	United States	Randomized, 2,000 participants	efficacy of hydroxychloroquine (HQ) and azithromycin (Azi)	Phase 2	Expected by March 5, 2021	https://clinicaltrials.gov/ct2/show/NCT04358068
				Day 0: HQ 400 mg (200 + 200) + Azi 500 mg (250 + 250) orally			
				Day 1–6: HQ 200 mg (twice/day) + Azi 250 mg (4 days)			
3	NCT04333654 April 12, 2020	United States, Belgium, France, Netherlands	Randomized, 210 participants	HQ loading dose on day 1, maintenance dose till day 9	Phase 1	Expected by August 2020	https://clinicaltrials.gov/ct2/show/NCT04333654
4	NCT04358081May 1, 2020	United States	Randomized, 444 participants	HQ monotherapy (600 mg) and in combination With Azi (200 mg)	Phase 3	Expected by July 24, 2020	https://clinicaltrials.gov/ct2/show/NCT04358081
				HQ (600 mg) with or without Azi (500 mg)			
5	NCT04381936 (March 19, 2020)	United Kingdom	Randomized, 12,000 participants	Oral dose	Stopped	No clinical benefit. Out of 1,542 patients administered with hydroxychloroquine, no significant difference in primary endpoint of 28-days mortality. (25.7% HQ as compared with 23.5% usual care alone)	https://clinicaltrials.gov/ct2/show/record/NCT04381936; https://www.recoverytrial.net/files/hcq-recovery-statement-050620-final-002.pdf
				Initial: 800 mg, 6h: 800 mg, 12h:400 mg, 24h: 400 mg, every 12h thereafter for 9 days: 400 mg			
6	NCT04308668 (March 17, 2020)	United States, Canada	Randomized, 3,000 participants	Oral dose 200 mg tab	Phase 3	After high or moderate risk exposure to COVID-19, HQ did not prevent illness when used as postexposure prophylaxis within 4 days after exposure	https://clinicaltrials.gov/ct2/show/NCT04308668
				Initial: 800 mg orally once 4 days: 600 mg (every 6–8 h)			
7	NCT04304053 (March 18, 2020)	Spain	Randomized, 2,250 participants	Testing, treatment and prophylaxis of SARS-CoV-2	Phase 3	No significant results	https://clinicaltrials.gov/ct2/show/NCT04304053; https://www.sciencemag.org/news/2020/06/three-big-studies-dim-hopes-hydroxychloroquine-can-treat-or-prevent-covid-19
				Oral dose 200 mg tabs			
				Day 1: 800 mg			
				Day 2–7: 400 mg			
				Contacts			
				Day 1: 800 mg			
				Day 2–4: 400 mg			
8	NCT04303507 (April 29, 2020)	Thailand, United Kingdom	Randomized, 40,000 participants	Prophylaxis Study Loading dose: 4 tabs of 155 mg/60 kg body weight 90 days: 155 mg daily	Not mentioned	Expected by April 2021	https://clinicaltrials.gov/ct2/show/NCT04303507

The studies by Patel and coworkers (https://www.sciencemag.org/news/2020/06/mysterious-company-s-coronavirus-papers-top-medical-journals-may-be-unraveling; Mehra et al., 2020a; Mehra et al., 2020b), claimed to have performed a multinational registry analysis using a cloud-based health-care data analytics platform, Surgisphere Corporation, Chicago, IL, United States, on the usage of hydroxychloroquine or chloroquine with or without a macrolide for the treatment of COVID-19. They reported an increased risk of in-hospital mortality and de-novo ventricular arrhythmia in response to the treatment which led to the inference that hydroxychloroquine or chloroquine, when used alone or with a macrolide does not offer any benefit to the COVID-19 patients, which contributed to the halt in worldwide clinical trials by the WHO on May 25, 2020. The second study (Mehra et al., 2020b) claimed to negate the association of ACE inhibitors and angiotensin-receptor blockers (ARBs) with in-hospital COVID-19 deaths. Their analyses brought forth better survival rates due to the use of either ACE inhibitors or statins. However, the authors mentioned that since the study was not based on randomized trials, there could be a possibility of confounding and hence, concluded that an underlying cardiovascular disease is independently associated with an increased risk of in-hospital COVID-19 death.

Substantive red flags were raised by the rattled global scientific community because the doses in the reported cases were higher than those set by the United States FDA and discrepancies in the official COVID-19 mortality statistics, and sample size (https://www.sciencemag.org/news/2020/06/mysterious-company-s-coronavirus-papers-top-medical-journals-may-be-unraveling). Eventually, both the studies were retracted from the journals, The Lancet and The New England Journal of Medicine. Currently, clinal trials in various parts of the world have resumed to investigate the potential use of hydroxychloroquine in COVID-19 patients in response to WHO’s green signal (https://www.sciencemag.org/news/2020/06/mysterious-company-s-coronavirus-papers-top-medical-journals-may-be-unraveling). In another report by Geleris et al. (2020) reported no positive or negative observational effect of hydroxychloroquine on death or incubation risk on COVID-19 patients, however, this study did support the further randomized clinical trials of hydroxychloroquine testing its efficacy.

The United Kingdom’s mega RECOVERY trial (RECOVERY Collaborative Group, 2020) reported the ineffectiveness of hydroxychloroquine. The patients who received the treatment demonstrated a longer hospitalization duration, higher risk of mechanical ventilation or mortality than those who received the usual care. However, the study received sceptical reviews due to the high dosage issues: 800 mg at 0 and 6 h, followed by a 400 mg dose at 12 h and every 12 h thereafter for 9 days; which may have contributed to cardiovascular, neurological, and other toxicities. The authors chose this dosage based on extensive pharmacokinetic studies.

On December 2nd, 2020, the WHO Solidarity Trial Consortium published the findings of their trials on repurposed anti-viral drugs for COVID-19 (NCT04315948). The drugs included hydroxychloroquine, remdesivir, lopinavir, and interferon beta-1a in hospitalized COVID-19 patients. The randomized trials were evaluated for death rate according to age and requirement of mechanical ventilation. Like the RECOVERY trials, this one too reported negligible effect on mortality, ventilation, and hospitalization duration of COVID-19 patients (WHO Solidarity Trial Consortium, 2020).

## Points to Be Considered in the Current Pandemic Time With Chloroquine as a Therapeutic

Several ongoing clinical trials against COVID-19 with chloroquine/hydroxychloroquine alone or in a combination of drugs are the outcome of promising *in vitro* results and the hype created worldwide over the drug (Cortegiani et al., 2020). Giving too much of attention by the scientific community generates false promises and hampers the path of other potential drugs against COVID-19 in this pandemic era. Simultaneously, no negative feedback against chloroquine should be postulated without confirming the clinical trial results. Chloroquine being an age-old drug, has already been used against multiple diseases. If found effective, its inexpensive nature and already documented toxicity profile and dosage optimization can save time and a million lives. Though mixed results of chloroquine against COVID-19 have been obtained so far, there is an urgent need to test their effects and toxicity as a prophylactic drug, in mildly ill patients and severely ill patients of COVID-19. Moreover, one should never forget the thin line between chloroquine as a therapeutic agent and chloroquine poisoning (Kelly et al., 1990). An in-depth toxicity analysis of chloroquine and derivatives is required before confirming any comment for/against its use in time of COVID-19. The poor methods of clinal trials and its reporting has thus far been inadequate in proving the effective nature of hydroxychloroquine. Ferner and Aronson (2020) claim that the overuse of hydroxychloroquine will result in rare buy harmful cutaneous adverse reactions, hepatic problems and ventricular arrhythmias when prescribed in combination with azithromycin. In a recent study, Haque et al. (2021) reported the changes in purchasing patterns and pricing of hydroxychloroquine since March 2020 in India states. While no price and utilization changes were observed, hydroxychloroquine shortages were encountered due to the misinformation and management of COVID-19.

Among the rapidly changing guidelines in this pandemic era of COVID-19, WHO has revoked the ban on clinical trials with chloroquine against COVID-19 (https://www.sciencemag.org/news/2020/06/mysterious-company-s-coronavirus-papers-top-medical-journals-may-be-unraveling), however, FDA has cautioned its use outside of the hospital setting or a clinical trial due to risk of heart rhythm problems (https://www.fda.gov/drugs/drug-safety-and-availability/fda-cautions-against-use-hydroxychloroquine-or-chloroquine-covid-19-outside-hospital-setting-or). Though the preventive or prophylactic potential of chloroquine and the derivatives are yet to be confirmed against COVID-19, extensive, collaborative, unbiased and random clinical trials are required instead of small and individual trials to conclude. Results of unprejudiced, statistically significant and ethical outcomes of clinical trials are eagerly awaited before sealing the fate of this age-old drug against COVID-19.

## Conclusion and Future Aspects

The “age-old” drug used to treat multiple diseases has generated mixed therapeutic responses against “COVID-19.” Chloroquine has been recognized as a miracle medicine to treat malaria, autoimmune diseases, cancer, viral, dermatological, and fungal infections. Different *in vitro* and *in vivo* studies have suggested the positive, neutral, and negative role of chloroquine and derivatives against SARS-CoV-2. Though some studies are still ongoing, different probable mechanisms have been reported in literature employed by chloroquine to inhibit SARS-CoV-2 infection or cause more harm than good. In this difficult situation where an effective anti-viral drug is urgently needed, a biased decision against or in favour of chloroquine can either generate a false sense of security or can add more anxiety in an already worse situation. However, the previous research done on chloroquine against multiple diseases can help establish its anti-SARS-CoV-2 mechanism, precautions to be taken to avoid chloroquine’s toxicity, and dosage–optimization to reach any conclusion.
